# Anatomical Reasons for an Impaired Internal Jugular Flow

**DOI:** 10.3390/medicina61091627

**Published:** 2025-09-08

**Authors:** Viviana Dincă, Paris Ionescu, Răzvan Costin Tudose, Mădălin Munteanu, Alexandra Diana Vrapciu, Mugurel Constantin Rusu

**Affiliations:** 1 Division of Anatomy, Department 1, Faculty of Dentistry, “Carol Davila” University of Medicine and Pharmacy, 050474 Bucharest, Romaniarazvan-costin.tudose0721@stud.umfcd.ro (R.C.T.); mugurel.rusu@umfcd.ro (M.C.R.); 2 Department of Obstetrics and Gynaecology, Ovidius University, 900527 Constanţa, Romania; 3 Research Department, “Dr. Carol Davila” Central Military Emergency Hospital, 010825 Bucharest, Romania; 4 Institute for Cardiovascular Diseases of Timişoara, Clinic of Anesthesia and Intensive Care, “Victor Babeş” University of Medicine and Pharmacy Timişoara, 300310 Timişoara, Romania

**Keywords:** atlas vertebra, styloid process, Eagle syndrome, jugular vein, carotid artery, neck veins, inferior petrosal sinus, anatomical variation

## Abstract

The internal jugular vein (IJV) is of utmost importance during various surgical and endovascular approaches, including central access. It descends through the parapharyngeal space, carotid triangle, and sternocleidomastoid region. The anatomical variables of the IJV are mainly related to its calibre and dominance, number of venous channels (i.e., duplications and fenestrations), and compression sites. Specific compressions of the IJV are not exclusively due to the jugular nutcracker between the styloid process (SP) of the temporal bone and the C1 transverse process, which, in turn, should not be granted the eponym of Eagle. The possible morphologies of the SP and ossified stylohyoid chain are discussed here. Additionally, the digastric and sternocleidomastoid muscles, the hyoid, and the distorted carotid arteries may compress the IJV, thereby raising intracranial pressure. Here, a case is documented with a long inferior petrosal sinus adjacent to the IJV, both compressed into the C1–styloid nutcracker, which is an absolute novelty. Multiple compression sites of the IJV are supported here with original evidence. All anatomical variables of the IJV are relevant, as they may lead to stenoses or interfere with IJV cannulation. In rare cases of IJV agenesis, multiple compression sites on the opposite side may significantly alter bilateral cerebral drainage. Different methods may be used to decompress a stenotic IJV, including styloidectomy. In conclusion, the anatomical variables of the IJV should be acknowledged by practitioners and documented on a case-by-case basis.

## 1. Introduction

The internal jugular vein (IJV) is a major blood vessel of the neck, of utmost importance for anaesthesiologists, surgeons, and anatomists [1]. It drains the sigmoid sinus into the brachiocephalic (innominate) vein. Cannulation of a central vein is an everyday procedure in anaesthesiology [2,3,4].

Internal jugular vein stenosis is receiving increased attention from the medical research community [5]. The condition causes multiple confusing symptoms that negatively impact patients’ daily lives [5]. These symptoms remain unexplained by other established diagnostic categories [5].

This review addresses an important and underexplored topic, namely, the anatomical variations and compression sites of the IJV, with potential clinical implications. They are usually approached independently; thus, the integrative anatomical picture may have been overlooked.

## 2. Normal Anatomy of the Internal Jugular Vein

The IJV continues the sigmoid sinus in the jugular foramen, located just above the anterior surface of the transverse process of the atlas [6]. The uppermost part of the IJV receiving the sigmoid sinus is the jugular bulb [7]. The anterior condylar confluent of Trolard, or the petrosal confluence, lies at the external opening of the hypoglossal canal, immediately antero-inferior to the jugular bulb [8,9,10]. The confluent of Trolard is connected directly with the IJV, inferior petrosal sinus (IPS), anterior condylar vein, inferior petroccipital vein, lateral condylar vein [9], prevertebral vein [11] or plexus [12], and, eventually, clival diploic veins [13]. These may serve as collateral pathways, at least partly, to divert the flow from a compressed IJV.

The IJV continues in the poststyloid part of the parapharyngeal space [14]. It then courses in the carotid triangle. There, it runs at the anterior margin of the sternocleidomastoid (SCM) muscle. In the lower neck, the IJV is positioned deep to the SCM muscle. It participates in the cervical vasculonervous bundle. This bundle is contained inside the carotid sheath. At the level of the sternoclavicular joint, the IJV joins the subclavian vein. This junction occurs at Pirogoff’s jugular angle. Together, they form the brachiocephalic (innominate) vein. The IJV has marked variability in its cervical course [15]. It projects onto a band joining the ear lobe and the sternoclavicular joint [16]. In the lower neck, the IJV courses adjacent to the common carotid artery (CCA). The IJV is a common route for percutaneous placement of a venous cannula for measuring central venous pressures and pulmonary pressures [17,18].

The IJVs constitute the primary outflow route from the head and neck in humans [19]. In the supine body position, most of the blood from the head flows through the IJVs [19,20,21,22]. Still, in the upright posture, the IJVs collapse, and the venous outflow is directed via the posterior neck veins, the suboccipital plexus, the vertebral plexus and veins, the epidural veins, and the deep cervical veins [19,20,21,22].

An interesting study on 100 computed tomography (CT) angiograms found that as the IJV descends into the thorax, its area increases and it geometrically resembles a conical structure [23]. The problem with comparing a cross-sectional area at one level of the IJV to the largest cross-sectional area in the conical IJV is that the intrinsically small cross-sectional area of the more cranial levels will mathematically appear stenotic compared with the larger, flangelike cross-sectional area of the caudal IJV [15].

The left IJV is smaller than the right IJV in one third of adults, this being statistically significant [15,23,24,25]. No correlation was found between the IJV diameter and subject height, weight, age, or neck circumference [26].

The retrostyloid compartment of the upper parapharyngeal space comprises the internal carotid artery (ICA), IJV, cranial nerves IX to XII, and the superior sympathetic trunk [27,28]. The wall of the IJV is thinner than that of the ICA, and the lesions in the upper parapharyngeal space often compress it [27]. In cadavers, the IJV was found at the posteromedial and posterolateral aspects of the styloid process (SP) in 13 (65%) and 7 (35%) sides, respectively [27].

Compression of the IJV is a common and clinically significant condition, most often caused by nearby bones or muscles. It can lead to a range of neurological symptoms and, in severe cases, serious complications. Surgical and endovascular treatments are effective for symptomatic cases. Intentional IJV compression is a promising area for preventing brain injury [29,30,31], but further research is needed to confirm its safety and efficacy.

The most commonly identified site of outflow impairment is in the rostral IJV near the transverse process of C1 and the SP, often referred to as styloidogenic jugular stenosis [32].

IJV compression may cause venous reflux. This can alter cerebral drainage patterns. These changes affect cerebrospinal fluid dynamics. The compression can be detected using several methods. Doppler ultrasound is one option. MRI and CT venography are also effective. Dynamic imaging during head movement may reveal positional compression. IJV compression is relevant in various medical contexts. It can explain specific headache patterns. It also helps us understand cerebral venous drainage disorders. However, some aspects remain unclear. The clinical significance of mild IJV compression in asymptomatic individuals is uncertain. Some degree of positional variation in IJV patency is normal. The field continues to evolve. Imaging techniques are improving. Our understanding of cerebral venous drainage is becoming more sophisticated. An IJV compression can lead to venous shunting to the petrosal sinuses, which drain the inner ear, and to the vertebral veins of the cervical spinal cord, resulting in symptoms referable to the areas of shunting [33,34].

### 2.1. The Segments of the Internal Jugular Vein

Three segments of the IJV are defined for ultrasonographic measurements. These segments are used during various examinations. The first is the inferior segment (J1). This extends from the confluence of the IJV with the subclavian vein. It reaches the inferior level of the thyroid gland. The second is the middle segment (J2). This starts from the inferior level of the thyroid gland. It continues to the jugular point where the IJV crosses the carotid bifurcation. The third is the superior segment (J3). This begins from the jugular point. It extends to the highest ultrasonographically detectable point of the IJV [35,36,37,38]. Suppose one takes into consideration that the vertical level of the carotid bifurcation is individually variable [39]. In that case, this also means that the jugular point glides vertically from case to case, and thus, the lengths of the J2 and J3 segments oscillate.

We consider that these divisions should be indicated on an anatomical basis, from the base of the skull to the base of the neck. Indicating that the J1 segment is the proximal portion of the IJV and the subclavian/brachiocephalic vein, the J2 segment is the IJV’s segment at the level of the thyroid gland, and the J3 segment is at the level of the carotid bifurcation, as we found in a recent study [38], may leave the upper, parapharyngeal segment of the IJV unclassified.

As the J3 segment of the IJV passes through the “tunnel” between the transverse process of the atlas and the SP, this segment is more likely to be impinged by osseous structures from cervical spondylosis [40].

### 2.2. The Internal Jugular Vein’s Valves

The IJV valves make a buffer zone between the large central veins and the cerebral venous system [41]. The valves are generally located about 0.5 cm above the union of the subclavian vein and the IJV, in 96.8% of the general population [41]. The IJV valves prevent the backflow of venous blood and backwards venous pressure into the cerebral venous system during conditions where the central venous pressure or intrathoracic pressure is increased, such as chest compression during external cardiopulmonary resuscitation, severe or repetitive cough, and straining [41]. Without competent IJV valves, a sustained or prolonged retrograde-transmitted venous pressure via IJVs might impair cerebral venous drainage and determine neurological deficits, such as encephalopathy, after cardiopulmonary resuscitation [41]. It was previously reported that the valves are usually bicuspid, sometimes unicuspid, and rarely tricuspid, with inconsistent cusp orientation, raising questions about their functional efficacy, particularly in limiting competence to foetal life [42].

### 2.3. The Dominant Internal Jugular Vein

A dominant IJV refers to the vein (either right or left) that is significantly larger in cross-sectional area compared to the opposite side, often defined as at least twice the size of the contralateral vein. This dominance is significant because it typically serves as the primary pathway for venous blood drainage from the brain. Asymmetry between the IJVs is common, with one side being dominant in over 60% of individuals. The right IJV is dominant in most cases, but not exclusively so [43,44]. Jayaraman et al. (2012) reviewed 108 cases and found that in 49.1%, the right IJV was dominant, while in 19.4%, the left IJV was dominant, and in 31.5%, the IJVs were co-dominant [45]. The size and dominance of the IJVs can vary widely between individuals and populations, which is relevant for both surgical planning and diagnostic procedures [44].

### 2.4. The “Tunnel” of the Internal Jugular Vein at the Level of the Transverse Process of the Atlas

**Figure 1:**
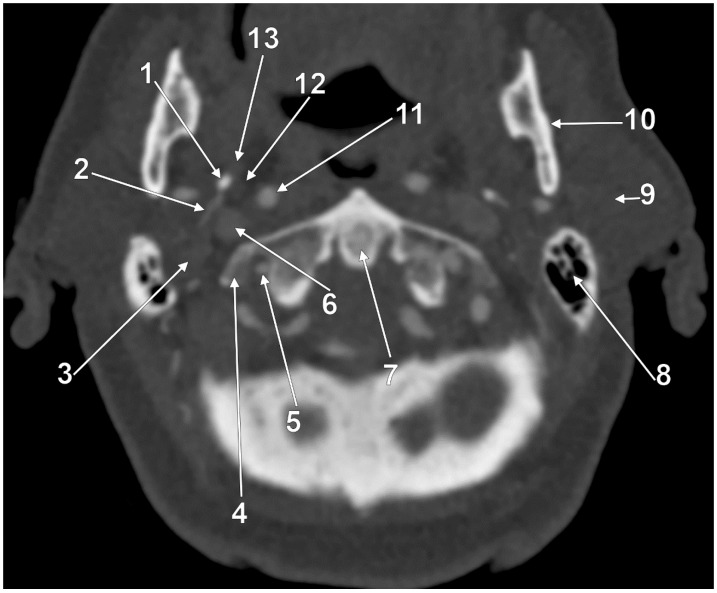
Axial angioCT slice through the “tunnel” of the internal jugular vein at C1. Inferior view. Original evidence. 1. styloid process; 2. occipital artery; 3. posterior belly of the digastric muscle; 4. transverse process of the atlas; 5. vertebral artery; 6. internal jugular vein; 7. dens axis; 8. mastoid process; 9. parotid gland; 10. mandibular ramus; 11. internal carotid artery; 12. stylopharyngeus muscle; 13. styloglossus muscle.

**Figure 2:**
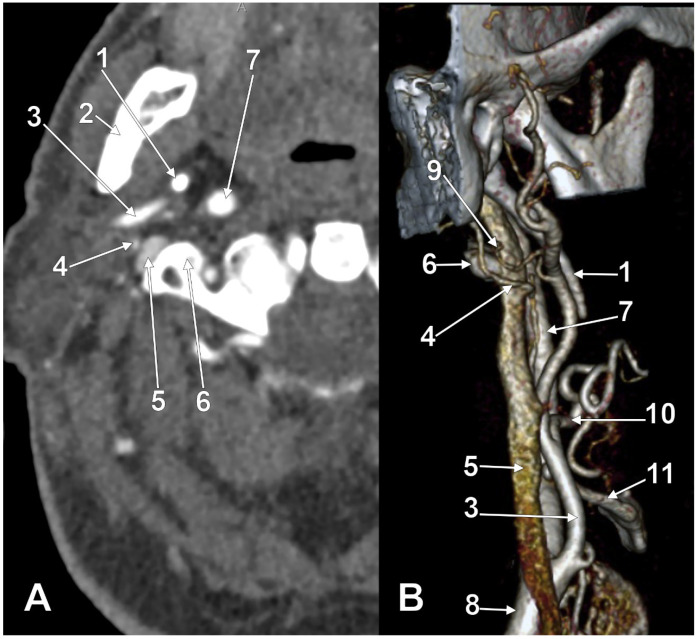
The retrostyloid course of the external carotid artery brings it anteriorly to the internal jugular vein. Right side. Original evidence. (**A**) Axial slice at the level of the transverse process of the atlas, inferior view. (**B**) Three-dimensional volume rendering, posterolateral view. 1. styloid process; 2. mandibular ramus; 3. external carotid artery; 4. occipital artery; 5. internal jugular vein; 6. transverse process of the atlas; 7. internal carotid artery; 8. common carotid artery; 9. posterior auricular artery; 10. linguofacial trunk; 11. greater hyoid horn.

At C1, the IJV descends through a veritable vertical tunnel bordered by different anatomical structures, each of these having the potential to compress the IJV. The tunnel of the IJV is limited: posteriorly, by the C1 transverse process, through which courses the vertebral artery, anteriorly, by the SP, medially, by the ICA, antero-medially, by the stylopharyngeus and styloglossus, laterally, by the posterior belly of the digastric muscle, the SCM, and, eventually, the mastoid process, and anterolaterally, by the deep lobe of the parotid gland and the external carotid artery (ECA) (Figure 1). A retrostyloid course of the ECA [46] will bring it between the SP and the IJV (Figure 2).

At this level, the IJV is usually crossed laterally by the occipital (OA) and posterior auricular (PAA) arteries (Figure 2). The OA may, however, occasionally cross the medial side of the IJV [47]. On the IJV, the deep cervical lymph nodes of the jugular chain are applied, the most representative of these being the subdigastric ganglion or Küttner’s main ganglion [48].

## 3. Anatomical Variations of the Internal Jugular Vein

Anatomical variations of the IJV are uncommonly reported, and most of the reports are from anaesthetists and are based on evaluation of imaging for central venous access [16]. Knowledge of the anatomical variation in IJV anomalies may be critical to avoid iatrogenic injury [49]. The variants of the IJV can modify the surgical routes or landmarks related to the IJV [50].

Variations in IJV anatomy are not always pathologic; however, when symptoms of intracranial hypertension are observed, further investigation is warranted [51]. The jugular bulb may lie exposed within the middle ear cavity; however, there are different morphological variations of the jugular bulb [7,52], as listed in Table 1. The variations of the IJV include trifurcation (0.08%), bifurcation (0.33%), duplication (1.17%), fenestration (1.33%), and posterior tributary (0.41%) [16]. Fenestrations of the IJV can be partial or complete and may occur at different heights [50,53]. The external branch of the accessory nerve passes anterior to the IJV (70%), posterior to it (26.8%), or through a fenestrated or duplicated IJV [52].

IJV agenesis represents an extremely uncommon congenital anomaly that is usually clinically silent and identified incidentally on imaging performed for other clinical reasons [50,54]. The contralateral IJV may sometimes develop compensatory enlargement to handle the additional venous return from cerebral and cervical structures, potentially leading to vessel dilation [50]. Nevertheless, although the IJV may be present, the sigmoid sinus on that side may be absent or thread-like [55,56]. Thus, the opposite IJV may compensate for the abnormal contralateral sigmoid sinus. Therefore, compression of the compensatory IJV is a major risk factor for cerebral drainage.

**Table 1:** Jugular bulb morphological variations overview. JB: jugular bulb; IAC: internal acoustic canal; PSC: posterior semicircular canal; JBD: jugular bulb diverticulum; HJB: high jugular bulb.

Jugular Bulb Variant	Definition	Clinical Significance	Prevalence/Range	References
High JB	JB apex abnormally high vs. otic landmarks (e.g., reaches/traverses IAC floor; above round window/basal turn/superior annulus). Morphology: “waist-like” JB (vs. finger-like diverticulum).	Can narrow Trautmann’s triangle; bleeding risk in otologic/skull-base work; common cause of pulsatile tinnitus.	Prevalence varies by definition/method, ranging from ~0% to 46% across studies.	[57,58,59,60,61,62,63]
Dehiscent JB	JB extends into the middle ear via a dehiscent sigmoid plate; mucosa only (no complete bony cover).	Injury-prone during ear surgery; haemorrhage; associations with thin carotid canal wall/dehiscence.	Reported ~1–7.5%; side bias often right-dominant.	[63,64,65,66,67,68]
JB Diverticulum	True outpouching from the JB dome (often finger-like) projecting superiorly/medially/posteriorly within the petrous bone.	Pulsatile tinnitus; conductive/±sensorineural loss; vertigo; can complicate middle ear/skull-base routes.	Reported from ~0.8% up to ~8% (method/definition dependent).	[58,60,62,63,67,69,70]
Hypoplastic JB	Proposed threshold: ≤5 mm max diameter.	May reflect reduced venous pathway calibre; may be consistent in measurement plane/modality.	Not standardised; threshold proposed to normalise reporting.	[71,72,73]
Hyperplastic JB	Proposed threshold: ≥15 mm max diameter.	Consider high-flow states/bone disorders; evaluate adjacent bony walls.	Not standardised; noted with achondroplasia/Paget’s disease, etc.	[71,72,74]
Topographic Variants	Type 1: none; Type 2: below PSC; Type 3: between PSC and IAC; Type 4: above IAC; “A” intact bone, “B” dehiscent.	Guides risk mapping near PSC/IAC; flags dehiscence (“B”) zones.	Classification, no pooled prevalence.	[69,75,76]
Condylar Jugular Diverticulum	Actually an enlarged condylar canal communicating with JB; best seen on posterior coronal images (maintains calibre to condylar fossa).	Prevents mislabelling as JBD and unnecessary intervention.	—	[60,70,77]

**Figure 3:**
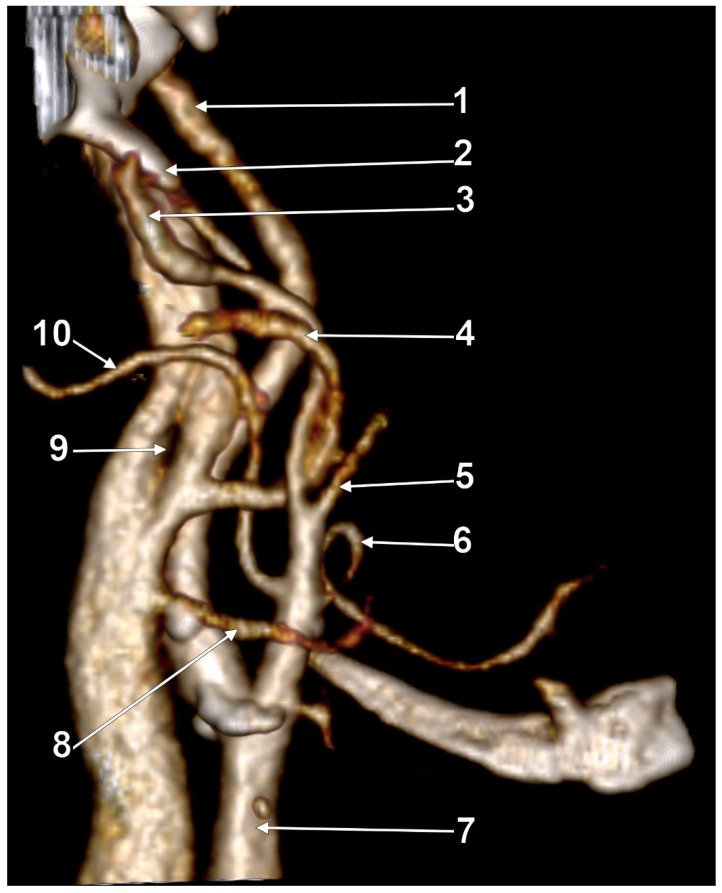
Fenestrated right internal jugular vein, with the anterior arm of the fenestration receiving the right external carotid vein (Launay’s vein). Original evidence. Three-dimensional volume rendering. Right anterolateral view. 1. internal carotid artery; 2. styloid process; 3. external carotid artery; 4. Launay’s external carotid vein; 5. facial artery; 6. lingual artery; 7. common carotid artery; 8. lingual vein; 9. fenestration of the internal jugular vein; 10. occipital artery.

One of the arms of a fenestrated IJV may receive the external carotid vein of Launay (Figure 3). The vein of Launay has different morphological and topographical possibilities; it empties into the IJV and may connect with an opposite one via the prevertebral plexus [78,79]. Therefore, the prevertebral plexus (anterior external vertebral venous plexus) may be used to divert the flow from a compressed IJV to the opposite, compensatory one, when the vein of Launay is bilateral.

The posterior tributary of the IJV is unusual and is scarcely reported in the literature—just three cases were documented by Mumtaz and Singh in 2019 [16]. Two other cases were reported by Hage et al. in 2024 [80].

The causes of non-compression chronic cerebrospinal venous insufficiency include the high jugular bulb (77.27%), fenestration of the IJV (7.27%), IJV ectasia (2.73%), and tortuous IJV (0.91%) [81]. The IJV ectasia is an underreported anomaly of the vein [82]. It may become visible when performing the Valsalva manoeuvre [82]. As IJV ectasia is a dilated segment of the vein, it can be differentiated from an enlarged segment proximal to a compression site. However, when multiple compression sites of the IJV exist, the enlarged segments between them have to be differentiated from ectatic sites without compression.

### Absence of the Internal Jugular Vein: Clinical Rarity, Causes, and Implications

Non-visualisation of a right IJV on ultrasonography can be due to operator (e.g., suboptimal patient positioning, inappropriate machine setting, or excessive pressure on the probe) and patient (e.g., vascular thrombosis, congenital anomalies such as hypoplasia or agenesis) factors [83].

**Figure 4:**
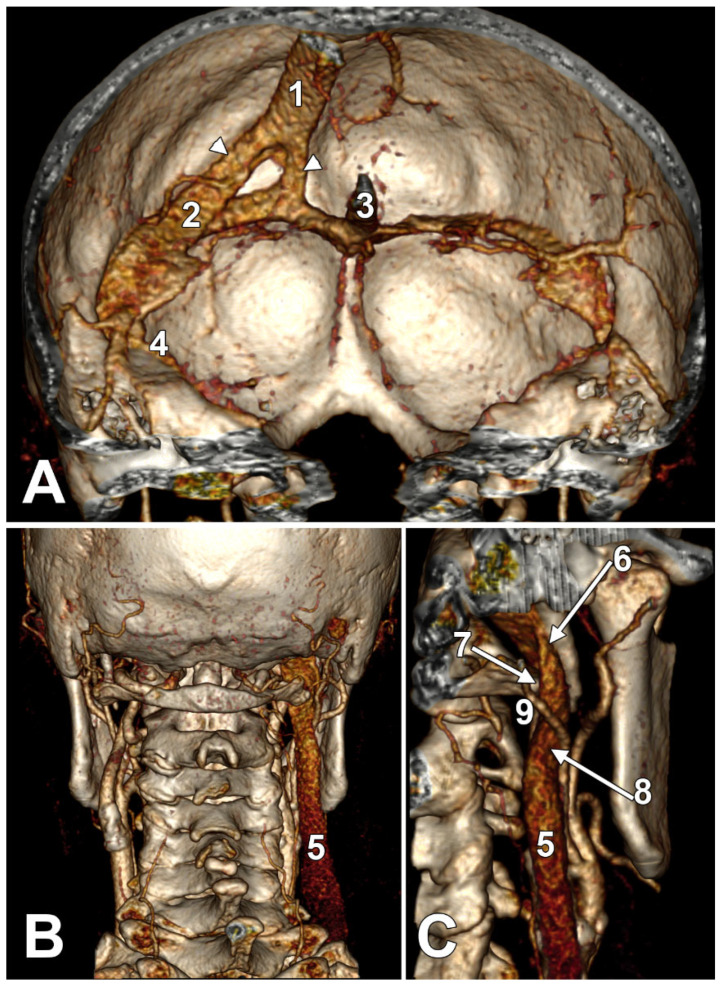
Agenesis of the left internal jugular vein, compensated via the right sigmoid sinus and internal jugular vein. Three-dimensional renderings: Original evidence. (**A**), Anterior view of the posterior cranial fossa; (**B**), posterior view of the skull and spine; (**C**), posterolateral view of the right internal jugular vein. 1. Bifid (arrowheads) superior sagittal sinus; 2. right transverse sinus; 3. straight sinus; 4. right sigmoid sinus; 5. right internal jugular vein, with three successive compression sites: anterior compression by the styloid process (6), posterior compression by the C1 transverse process (7), and lateral compression by the digastric muscle (8); 9. occipital artery, coursing deep to the posterior belly of the digastric muscle.

The absence of the IJV is an extremely rare anatomical anomaly [1], most often discovered incidentally during imaging or procedures, such as central line placement attempts. Awareness of this condition is crucial for clinicians to avoid complications during central venous access or neck surgery. This uncommon variation must be kept in mind while performing a central venous cannulation, especially if no ultrasound guidance is available [84]. In such cases of IJV agenesis, it is wise to avoid a cannulation attempt on the compensatory side to prevent future possible complications due to the alteration of both IJVs [84]. If the compensatory IJV is subjected to compressions (Figure 4), the clinical picture may complicate a cerebral insufficiency syndrome.

Congenital agenesis (true absence) of the IJV is very uncommon, with an estimated prevalence in the general population between 0.05% and 0.25% [85,86,87]. Most reported cases involve unilateral absence, typically discovered during imaging for unrelated conditions or failed attempts at venous cannulation [85,86,87,88]. Developmental arrest during embryogenesis is the primary cause of congenital absence or hypoplasia (underdevelopment) of the IJV [85,87]. Acquired absence can result from thrombosis, surgical removal, or trauma, but these are not true agenesis [86,87,89].

Patients are often asymptomatic due to compensatory enlargement of the contralateral IJV or collateral veins [85,86]. Enhanced drainage via the posterior neck veins or an increased flow through the external jugular veins may also compensate for the unilateral absence of the IJV. The drainage of intracranial blood from the side with an absent IJV could also be detoured via the transverse and sigmoid sinuses to the opposite side (Figure 4). Diagnosis is usually made via ultrasound, CT, or MRI, especially when the IJV cannot be visualised during central line placement [85,86,87,88]. Compensatory changes (e.g., enlargement of the opposite IJV) may mimic cervical masses [85,86,90].

The absence of the IJV can complicate or preclude cannulation; ultrasound guidance is strongly recommended [85,87,88,91]. Surgeons must be aware of possible IJV absence to avoid intraoperative complications, especially in oncological or reconstructive procedures [85,86,87]. Awareness of possible IJV agenesis is crucial during neck dissection surgery, requiring surgeons to be extremely careful to avoid accidental damage to the IJV on the unaffected side [54]. Recognition of this anomaly is essential before vessel ligation on the compensatory IJV, particularly in bilateral neck dissections [54]. Furthermore, surgeons must avoid disrupting compensatory venous drainage routes, including the external jugular system, which serves as an alternative pathway when venous agenesis is present [54]. Early detection of the IJV agenesis can help limit damage during medical therapy of neurological conditions, including headaches, and avoid misdiagnosis of other prevalent diagnoses [92].

## 4. The Styloid Process and the Stylohyoid Chain

The stylohyoid complex (SHC) consists of three key components, namely, the SP, the stylohyoid ligament, and the lesser horn of the hyoid bone, which derive from Reichert’s cartilage of the second pharyngeal arch [93,94,95]. This cartilage has four parts: tympanohyal, stylohyal, ceratohyal (keratohyal), and hypohyal [95]. Therefore, the SP consists of the tympanohyal and stylohyal parts; the stylohyoid ligament is the keratohyal or ceratohyal, while the lesser hyoid horn is the hypohyal part [96,97,98]. Calcification of the stylohyoid ligament (ceratohyal) may be associated with an autosomal dominant background, affecting between 4 and 28% of the population [99]. The SP attaches three muscles, the stylopharyngeus, styloglossus, and stylohyoideus, and two ligaments, the stylohyoid and stylomandibular ligaments, the latter being a condensation of the stylomandibular fascia. Orthopantomograms and CTs can be used to assess the SP/stylohyoid ligament complex [100].

Guimaraes et al. (2006) used a modified version of Langlais et al.’s (1995) classification of an elongated SP (ESP) and/or stylohyoid ligament ossification and found elongated, pseudo-articulated, and segmented SPs; they noticed that the morphological alterations of the SP develop in a symmetrical fashion [101,102]. However, although most SHC patterns are symmetric (80.4%), their asymmetry is not rare (19.6%) [103]. The pseudo-articulated type was found to have a pooled prevalence of 4.39%, and the segmented type was detected to have a pooled prevalence of 3.89% [104]. Different types of ossified SHC were also distinguished, namely, A, outline; B, partial; C, nodular; and D, complete [99]. Computed tomography is the most accurate method for detecting SHC variants [104].

The different patterns of ossification of the SHC (Table 2) result from a persisting Reichert’s cartilage that continues to grow and gradually ossifies into a bony chain; the joints in the chain often show some degree of bone clubbing, which implies a continuation of growth [96]. The patterns of ossification of the SHC were classified into three main types, depending on the number of bones in the trajectory of the hyoid apparatus [96]. The fundamental type consists of three bones: stylohyal, ceratohyal, and hypohyal (64%) [96]. In the major type A, the SHC has four bones: stylohyal, ceratohyal, accessory ceratohyal, and hypohyal (12%) [96]. In the primary type B, there are five distinctive bones: stylohyal, ceratohyal, accessory ceratohyal, accessory hypohyal, and hypohyal (12%) [96]. A restricted type with two bones was also described, which consists of the stylohyal and ceratohypohyal (fused cerato and hypohyal, 24%) [96].

**Table 2:** Main types of stylohyoid chain (SHC) variations.

Type/Pattern	Description	Prevalence/Notes	Citations
normal styloid process	typical length (<30 mm), no calcification	most common (74.97–86.5%)	[103,104]
elongated styloid process	length > 30 mm	7.1–25.03%	[99,103,104]
calcified stylohyoid ligament	partial or total ossification of the ligament	2.2% (partial more common than total)	[99,103]
segmented (pseudo-articulated) type	SHC appears as multiple ossified segments	3.89–4.39%	[104]
absent stylohyoid chain	SHC not visible or absent	2.8%	[103]

### The Length of the Styloid Process

The SP is a bony projection inserted onto the petrous bone just anterior to the stylomastoid foramen [105]. The length of the SP averages between 20 and 25 mm [105]. The right and left SP lengths do not show a statistically significant variation between females and males [105]. The unilateral absence of the SP was noted in 2.5% of cases [106].

Jung et al. (2004) observed that the SP is often denoted as elongated when it is longer than 30 mm or 33 mm [107]. After studying 1000 consecutive panoramic radiographs, these authors observed that the medians in that sample corresponded to the thresholds for ESP quoted in the literature [107]. However, these thresholds were too low, since they were exceeded in 50% of normal patients [107]. Therefore, they proposed that an ESP should be considered only if its length exceeds 45 mm, which corresponds to the average of the 90th percentiles for different sex and age groups [107].

Munoz-Leija et al. (2020) regarded a morphologically ESP as a continuous process of >25 mm [108]. Their results support the need for a new normal SP range, as the mean lengths of asymptomatic patients are above 30 mm; there is also a need to establish a standard range of angulation [108].

A longer SP has a greater movement capacity at its distal end, increasing the risk of adjacent neurovascular compromise [109]. As documented by Mantovani et al. (2023), in a physiological range of movement, during neck extension, the tip of the SP shows around 30 degrees of angular motion, and considering an SP length of 30 mm, the tip movement measures about 15 mm [109]. An ESP or vertically directed SP is prone to compress the IJV against the transverse process of the atlas [110,111,112].

An ESP is more common in older adults, with no correlation with gender [99,103,113]. Moreover, menopause does not affect the calcification or elongation of the SHC [113]. Symmetrical calcification patterns are more common, with 68.4% symmetry observed [93].

## 5. The Nutcracker Syndromes

The term “nutcracker” was first used in 1971 to describe the mesaortic compression of the left renal vein between the superior mesenteric artery and the aorta, this form being known as anterior nutcracker syndrome—the classic variant [114,115]. Posterior nutcracker syndrome involves compression of a retroaortic left renal vein between the aorta and the vertebral column [114,115,116]. Although this anomaly was initially discovered in 1950, it was not until 1972 that it received its specific name, “nutcracker syndrome.” This suggests that the term “nutcracker” refers to the compression of a vein between two arteries and/or bony structures.

According to several specialised articles, there is an association between Eagle jugular syndrome and jugular nutcracker syndrome (JNS) [109]. The first mention of Eagle syndrome (ES) appeared in 1937 [109]. However, at that time, the term “nutcracker” was not used in this context, indicating that there was no conceptual connection between the two terms.

## 6. Eagle’s Syndromes

In 1937, Watt W. Eagle reported two cases of ESPs [117]. Still, he credited Weinlecher for such a case observed in 1872 and for the first authentic report of clinical symptoms with subsequent removal of the SP [117]. According to Eagle (1937), ossification of the stylohyoid ligament had been recorded as early as 1652 by Demanchetis [117]. Later, Eagle classified two clinical syndromes due to ESPs: the typical syndrome and the carotid artery syndrome [118]. He emphasised that patients having typical symptoms due to an ESP are those who have undergone tonsillectomy and whose symptoms arise during post-tonsillectomy convalescence [118]. In the typical syndrome, “the characteristic symptoms are a constant pain or a nagging dull ache in the pharynx, presenting the sensation that the throat did not heal after tonsillectomy; a pain, frequently in the ear, indicating irritation of the vagus nerve; increased salivation; hesitancy and difficulty in swallowing; gagging; and a sensation of a foreign body—even cotton—in the pharynx” [118]. According to Eagle (1948), “The group of cases presenting the typical syndrome also includes all cases in which there is distortion of nerve function involving the sensory and motor fibers of the fifth, seventh, ninth, and tenth cranial nerves. The sensation of taste may be quite distorted. A spasm or contraction of the constrictor muscles of the esophagus and the pharynx may be observed. One patient having spasm of the constrictor muscles was caused much embarrassment each time he attempted to eat” [118]. However, subsequent studies have documented that an ESP can present with a myriad of symptoms, with no relation to tonsillectomy [119]. As for the carotid artery syndrome, Eagle described that in 1946, he had the first data to substantiate an idea he had entertained for six years that an ESP might produce pains along the carotid artery distribution [118]. He wrote the following: “since 1940 I had encountered patients, probably 12 in all, who had complained of pain beginning in the neck at a point about opposite the tonsillar fossa, which extended upward over the side of the head in a pattern conforming to the distribution of the ECA or the ICA. I had made notes that probably a carotid artery was involved in these cases in some way, but I had no definite evidence of this” [118]. It appears clear that Eagle did not refer to a syndrome specifically involving the compression of the IJV by an ESP. The ES were also referred to as “stylalgia” [120]. Confusion may appear if such erroneous descriptions are used in publications: “There are two distinct types of primary ES: primary stylo-carotid and stylo-jugular syndrome” [100].

**Figure 5:**
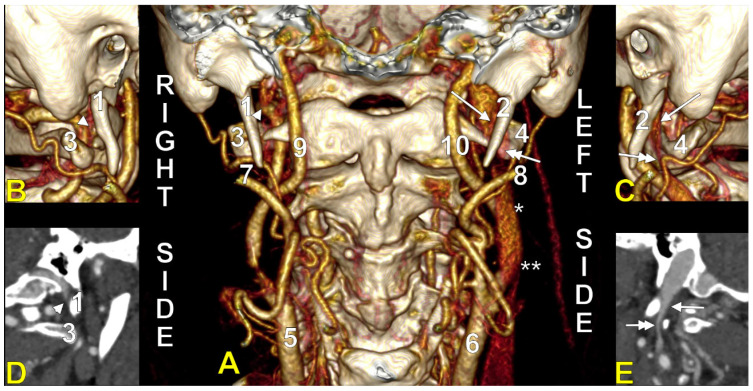
Posteriorly angulated styloid processes (SPs) (vertical angles < 27°) compress the internal jugular veins bilaterally. Original evidence. (**A**), Three-dimensional volume rendering, viewed anteriorly; (**B**), three-dimensional volume rendering, lateral view of the right SP; (**C**), three-dimensional volume rendering, lateral view of the left SP; (**D**), oblique sagittal slice through the right SP and the C1 transverse process, viewed laterally; (**E**), oblique sagittal slice through the left SP and the C1 transverse process, viewed laterally. 1. Right, bayonet-like SP; 2. left, straight SP; 3. right transverse process of the atlas; 4. left transverse process of the atlas; 5. right common carotid artery; 6. left common carotid artery; 7. right external carotid artery; 8. left external carotid artery; 9. right internal carotid artery; 10. left internal carotid artery. The arrowheads in (**A**,**B**,**D**) indicate the obstructed right internal jugular vein. The left internal jugular vein (**A**,**C**,**E**) has two upper sites of compression: by the SP (arrow) and, respectively, the C1 transverse process (double-headed arrow) and a double lower compression (*, **) by a posterior loop of the internal carotid artery.

The orientation and angle of the ESP determine which anatomical structures become compressed, resulting in various symptoms [98]. When deviated laterally, the process tip can compress the ECA at the point where it divides into the maxillary and superficial temporal arteries [98]. Posterior angulation (Figure 5) may entrap the ninth through twelfth cranial nerves, ICA, and IJV between the ESP and the C1 transverse process [98]. When the ESP deviates medially, it can impinge on the tonsillar fossa [98].

However, vascular variants of ES are rare [121]. The ESP length involved in vascular complications ranges from 31 to 77 mm, with an average length of 48 mm [121]. Vascular complications may involve either the IJV, ECA, or ICA [121].

ES is also referred to as “stylohyoid syndrome” [96]. Farina et al. (2021) classified ES into subtypes based on the structures that are compressed: classic jugular venous compression syndrome, stylo-carotid syndrome, and stylo-jugular venous compression syndrome [122]. However, this classification does not correspond to the two syndromes Eagle described initially. When these authors indicated that ES is caused by an ESP compressing the IJV, they quoted a publication by Suzuki et al. (2020), which reported “the first case of dural arteriovenous fistula in association with compressed IJV by an ESP” [123]. Suzuki et al. (2020) credited Zamboni et al. (2019) for defining “Eagle jugular syndrome” [123,124]. Indeed, in their Introduction, Zamboni et al. (2019) wrote that “a variant of the Eagle syndrome, where ESP is coursing adjacent to the transverse process of C1” and “often, in this anatomical situation, a significant compression of the IJV can be observed” [124]. However, Zamboni et al. (2019) noticed that few reports are available regarding symptoms and clinical presentation of such a “jugular vein bone nutcracker” [124]. While a JNS may be considered as describing the compression of the IJV between the SP and the transverse process of the atlas, the term “Eagle jugular syndrome” is a wrong attribution of the eponym. Mantovani et al. (2023) maintained the confusion of the eponym while stating that styloid jugular nutcracker syndrome is also known as Eagle jugular syndrome [109]. Moreover, they wrote that the styloid jugular nutcracker is also known as styloidogenic-cervical spondylotic IJV compression or styloid-induced IJV stenosis when describing it as an emerging pathological entity, variously related to several central nervous system disorders [109]. Other authors used “styloidogenic jugular venous compression syndrome” [111,125,126,127,128,129], a term that avoids the erroneous usage of Eagle’s eponym.

Cantin and Suazo Galdames (2011) quoted Camarda et al. (1989), who classified the cervicopharyngeal pain associated with an ESP into three distinct entities [130,131]. ES should be considered only when patients have no history of such an ESP before trauma, and the ossification of the stylohyoid chain develops in patients within a period after trauma, with accompanying symptoms [131]. A diagnosis of stylohyoid syndrome is applied only when patients have possible clinical and definite radiographic evidence of stylohyoid chain ossification and/or an ESP; there is no history of trauma, and the ossification has occurred during childhood or early adolescence without initial symptoms [131]. A pseudostyloid syndrome should be considered if there is neither a history of previous trauma nor clinical or radiographic evidence of ossification of the SHC [131].

It was observed that the juxtaposition of the SP and transverse process of the atlas can produce symptoms of cervicalgia and otalgia even in the setting of a normal-length SP [132]. Indeed, the elongation of the SP is not the only causative factor, but the process itself could present a specific angulation compressing the IJV [133].

## 7. Compressions of the Internal Jugular Vein

**Figure 6:**
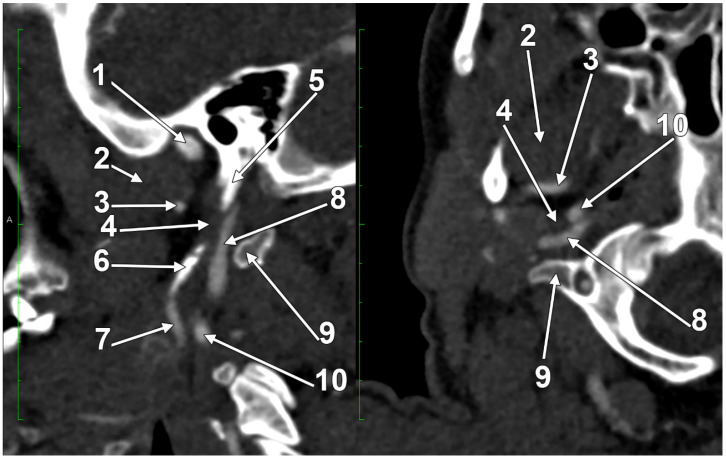
Correlated axial and oblique sagittal slices through the stylohyoid chain and the compressed segment of the internal jugular vein that demonstrates the tip pencil sign. 1. Medial pole of the mandibular condyle; 2. lateral pterygoid muscle; 3. maxillary artery; 4. stylohyoid ligament; 5. styloid process; 6. ossified stylohyoid ligament; 7. external carotid artery; 8. compressed internal jugular vein; 9. transverse process of the atlas; 10. internal carotid artery.

Any adjacent structure can cause direct compression of the IJV [134]. Extrinsic compressions of the IJV are somewhat due to anatomic variations rather than the ageing process [135]. The close anatomical relationship between the carotid artery system and the IJV may also explain why pathological changes in one can affect the other. The IJV can be compressed by various extrinsic anatomical structures and pathological conditions, which can have significant clinical implications. Areas of narrowing in the IJV greater than 50% occur most commonly in the upper cervical and skull base regions [15]. The leading anatomical causes of IJV compression are (1) SP compression: an ESP can compress the IJV and cause venous outflow obstruction and associated symptoms; (2) cervical spine and muscle compression: cervical spine abnormalities, including atlas (C1) and axis (C2) malformations, hypertrophied or spastic neck muscles, particularly the SCM; forward head posture can contribute to IJV compression; and (3) fascial and ligamentous structures: tight cervical fascia can restrict IJV expansion, anomalous fascial condensations or ligaments may also compress the vein. Compression of the IJV may be unilateral or bilateral and may be associated with collateral formation [45,136]. It was considered that, given the variability in the presence of these collaterals in unselected patients, it is unlikely that IJV stenoses are of physiologic significance because, in other regions of the body, venous stenoses are considered significant when collateral vessels are found [45]. On vertical slices, the IJV demonstrates the “tip pencil” sign [137], as in Figure 6.

When the IJV is compressed, several collateral venous routes can load and divert the flow of the IJV. In such situations, the enlarged compensatory veins are exposed to haemorrhagic risks during surgical procedures. Firstly, the contralateral IJV may enlarge and discharge the compressed vein’s dural suppliers (bilateral drainage through the IJV opposite to a compressed one). The superficial veins of the neck (external jugular, anterior jugular, and posterior external jugular) may redirect the flow from a compressed IJV via different communicating veins [138]. Thirdly, the IJV flow can be diverted through the veins of the suboccipital and posterior cervical regions, i.e., the deep cervical veins, either by direct connections or directly from the posterior cranial fossa via emissary veins [139,140]. Deep venous plexuses (pterygoid venous plexus, (para)pharyngeal venous plexus) are also anatomical alternatives to direct the flow through a compressed IJV.

Mandolesi et al. (2015), quoted in Piraino et al. (2018), classified chronic cerebrspinal venous insufficiency as three different types: (1) type 1, due to an endovascular obstacle, called hydraulic; (2) type 2, due to a muscular compression without endovascular anomalies, called mechanical; (3) type 3, presenting both endovascular and extravascular anomalies, called mixed; and (4) all three types [141,142]. Chronic cerebrospinal venous insufficiency may play a role in the pathophysiology of multiple sclerosis [143]. However, studies have not confirmed significant morphological or venous flow rate differences between patients with multiple sclerosis and controls [144].

Although contralateral rotation is the most crucial factor in obstructing the vein, some degree of extension may be required [6]. The addition of extension at the craniocervical junction to the position of the head causes the IJV, which is fixed in position at the jugular foramen, to be transported posteriorly in relation to the transverse process of the atlas, thus further increasing the compression associated with rotation [6].

A dissection study found that in all specimens, the posterior wall of the IJV rested against the transverse process of the atlas as the vein descended immediately below the jugular foramen [6]. In 38.8%, the transverse process of the atlas indented the posterior wall of the IJV, causing the vein to be slightly or moderately angulated as it descended across the anterior surface of the transverse process [6]. In 8.3% of the cases, the IJVs were severely kinked as they descended across the transverse process of the atlas [6]. Ding et al. (2020) observed that isolated C1 transverse process compression occurred more frequently in patients with unilateral IJV stenosis, whereas combined compression from both the C1 transverse process and styloid process was more likely to be found in cases of bilateral IJV stenosis [40].

The hypertrophy of the lateral mass of the atlas is a rare finding [145,146]. Specific references to hypertrophic C1 transverse process alone are limited. Therefore, for a jugular nutcracker, the transverse process of the atlas may be regarded as the fixed arm, while the SP, with variable length and angulation, may be regarded as the variable arm (Table 3).

**Table 3:** Common causes and outcomes of internal jugular vein compression.

Cause/Intervention	Main Outcome/Symptom	Reference(s)
Styloid process/C1 vertebra	Headache, tinnitus, insomnia	[45,128,133,147,148]
Muscular/osseous structures	Atypical facial pain, hypertension	[147,149]
Subcutaneous emphysema/tumour	Vein narrowing, variable symptoms	[150,151]
Compression collar (protective)	Reduced brain/axonal injury	[30,31,152,153,154]

On the other hand, external compression of the IJVs is an effective method for increasing intracranial blood volume and brain volume in animals and healthy humans [155]. It has been reported that, on assuming an upright posture, cerebral venous drainage is distributed away from the IJVs to the deep cervical veins/plexus [155]. Such intentional IJV compression (e.g., with a collar) prevents brain injuries by increasing the intracranial blood volume and reducing brain movement during trauma [30,31,152,153,154]. IJV stenosis is associated with several neurological disorders, including idiopathic intracranial hypertension (pseudotumor cerebri) and pulsatile tinnitus [156,157]. Such collars on the IJV may offer acute symptom relief for patients with venous pulsatile tinnitus [158]. In cases of extreme bony compression causing IJV stenosis, surgical decompression might be necessary [156].

IJV compression raises intracranial and intraocular pressures, increasing blood volume in the brain. This can reduce the movement (“slosh”) of the brain within the skull during impacts, potentially protecting against axonal injury and microstructural brain changes [152,153,159,160,161,162]. The use of IJV compression collars in athletes has been shown to reduce white matter alterations, preserve brain structure, and improve short-term neurocognitive outcomes following repetitive head impacts or concussions. However, these collars do not significantly reduce the overall incidence of concussion [154,159,161,162,163,164]. Laboratory studies using animal models revealed that IJV compression performed before traumatic brain injury or explosive blast exposure resulted in reduced neuronal damage, decreased brain bleeding, and mitigation of persistent hearing loss [30,31,152,153].

Jayaraman et al. (2012) investigated the causes of moderate or severe compression of the IJV, by side, in 108 cases [45]. On the right side, the compression of the IJV was determined by the SP in 24.1% of the cases, by the digastric muscle in 24.1%, by an artery (ICA, external carotid artery, or branches) in 2.8%, and by both the SP and digastric muscle in 4.6% of the cases [45]. On the left side, the respective prevalences were 30.6%, 11.1%, 1.8%, 0, and 0.9% [45]. We could not find details of the vertical levels of the IJV compressions determined by these authors or on the structure against which these elements compress the IJV. A case was reported in which the IJV was compressed not between the SP and the atlas, but between the SP and the rectus capitis lateralis muscle [165].

Sultan et al. (2022) reported a case with an ESP and concomitant compressions of the ICA and IJV [100]. These authors proposed a classification of the carotid and jugular compression syndromes due to an ESP: (1) Type IA: primary stylo-carotid syndrome with involvement of mid-ICA; (2) Type IB: primary stylo-carotid syndrome with involvement of proximal and mid-ICA; (3) Type II: primary stylo-jugular syndrome with impingement of the IJV; and (4) Type III: combined primary stylo-carotid–jugular syndrome.

Jugular (deep cervical) lymph nodes, located along the IJV, may also compress the vein [81,134,166,167]. Compression of the IJV by lymph nodes is most often associated with malignancy, particularly metastatic thyroid cancer and aggressive lymphomas (Table 4). The adenopathy compressing the IJV may, however, be reactive and not malignant [167]. While not the most common cause of IJV stenosis, the compression of the IJV by lymph nodes is clinically significant due to the risk of thrombosis and related complications. Early recognition and imaging are essential for appropriate management. While osseous (bony) compression is more common, lymph node compression accounts for a small but significant proportion of IJV stenosis cases (about 2.9% in a large Chinese cohort) [168]. The carotid arteries may also be medialised by massive lymphadenopathy [134].

**Table 4:** Table summarising cancers and mechanisms of IJV compression by lymph nodes.

Condition/Cancer Type	Mechanism of IJV Compression	Clinical Consequence	Citations
Thyroid cancer	Lymph node metastasis	Occlusion/thrombosis	[151,169]
Lymphoma (e.g., Grey Zone Lymphoma)	Mediastinal/cervical lymphadenopathy	Thrombosis, SVC syndrome	[170]
Gastric/prostate carcinoma	Metastatic lymphadenopathy	Thrombosis, embolic events	[171,172]

The proximity of the carotid axis and IJV within the carotid sheath makes the IJV susceptible to displacement when the carotid artery becomes tortuous. The normal anatomical relationship can be significantly altered, with the IJV being pushed away from its expected position. In 26.2% of cases, the ICA may present a more or less curved course to the base of the skull [173]. Curvature is somewhat more frequent in women than in men [173].

**Figure 7:**
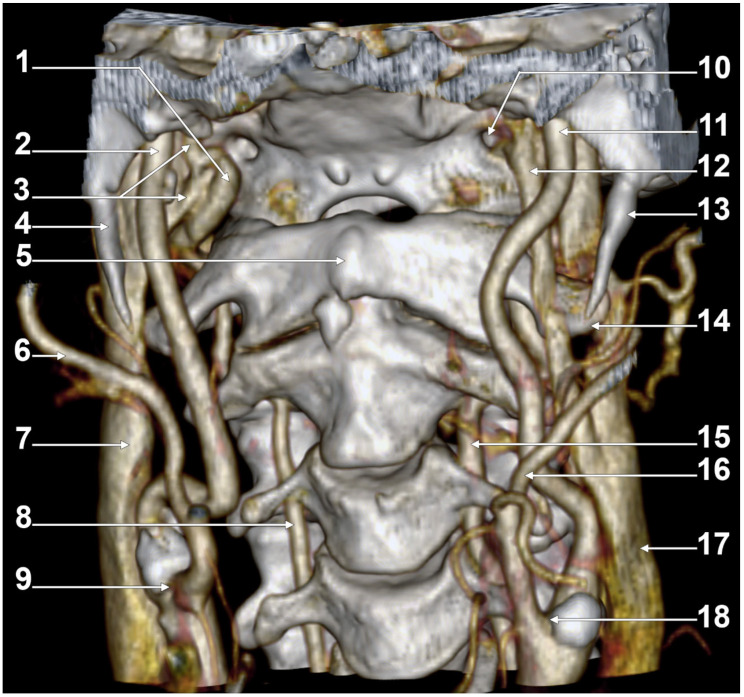
Bilateral long inferior petrosal sinuses of different lengths. Atheromatous origins of the internal carotid arteries are imprinted on the internal jugular veins. Three-dimensional volume rendering. Anterior view. 1. Right lateral condylar vein; 2. right internal carotid artery; 3. right inferior petrosal sinus; 4. right styloid process; 5. anterior arch of the atlas; 6. right external carotid artery; 7. right internal jugular vein; 8. right vertebral artery; 9. right carotid bifurcation; 10. left hypoglossal canal; 11. left internal carotid artery; 12. left inferior petrosal sinus; 13. left styloid process; 14. transverse process of the atlas (compression site); 15. left vertebral artery; 16. left external carotid artery; 17. left internal jugular vein; 18. left carotid bifurcation.

**Figure 8:**
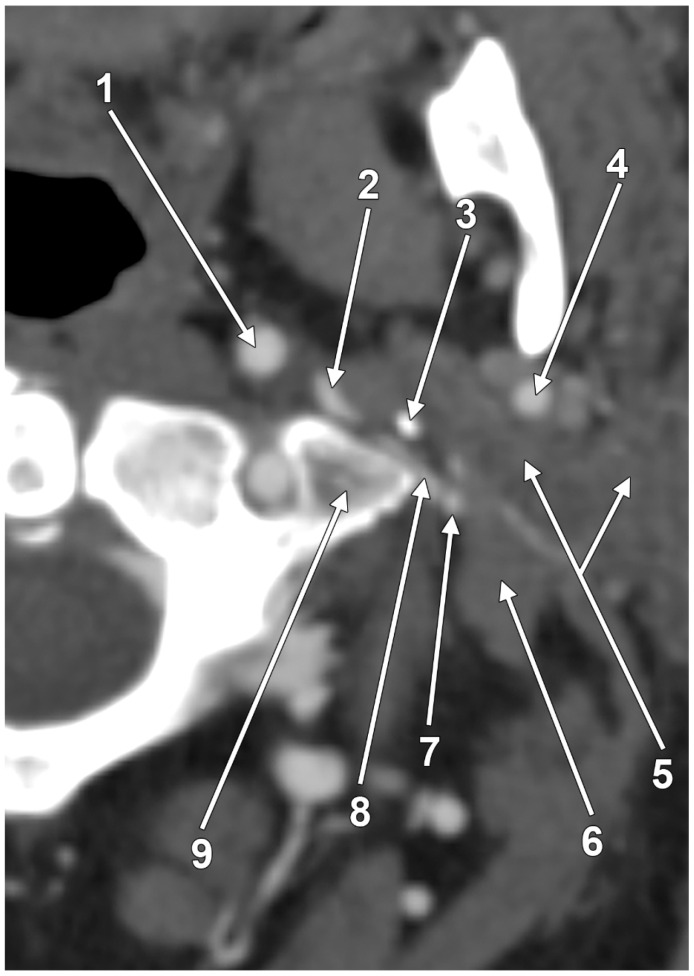
A long left inferior petrosal sinus and the internal jugular vein compressed on the transverse process of the atlas. Axial slice. Inferior view. Left side. 1. internal carotid artery; 2. inferior petrosal sinus; 3. styloid process; 4. external carotid artery; 5. parotid gland; 6. posterior belly of the digastric muscle; 7. occipital artery; 8. internal jugular vein; 9. transverse process of the atlas.

**Figure 9:**
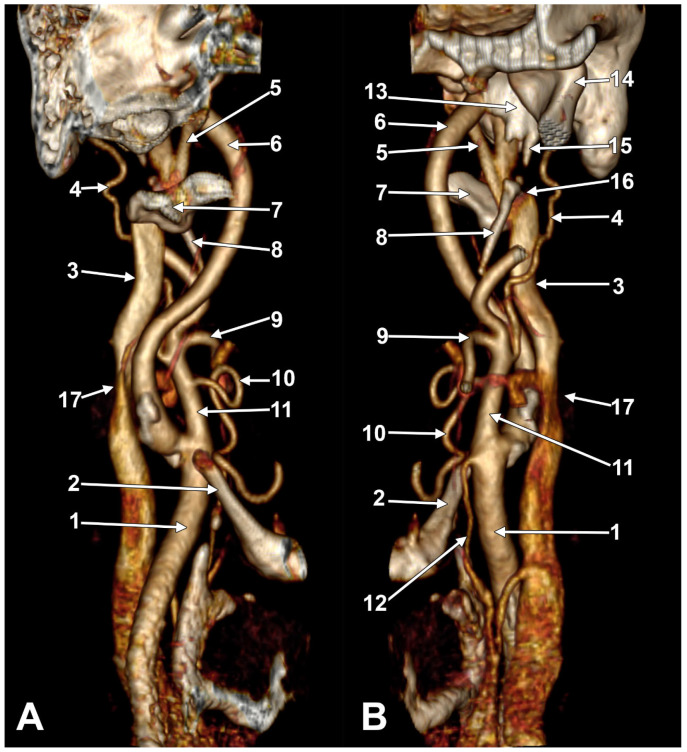
Triple compression sites of the internal jugular vein (IJV). Long inferior petrosal sinus. Left side. Three-dimensional volume rendering. (**A**) Antero-medial view; (**B**) anterolateral view. 1. Common carotid artery; 2. greater hyoid horn; 3. middle compression site of the IJV; 4. occipital artery; 5. extracranial segment of the inferior petrosal sinus; 6. internal carotid artery; 7. transverse process of the atlas; 8. ossified keratohyal; 9. facial artery; 10. lingual artery; 11. external carotid artery; 12. superior thyroid artery; 13. tympanic plate; 14. head of the mandible; 15. styloid process (stylohyal); 16. upper site of compression of the IJV; 17. lower site of compression of the IJV.

**Figure 10:**
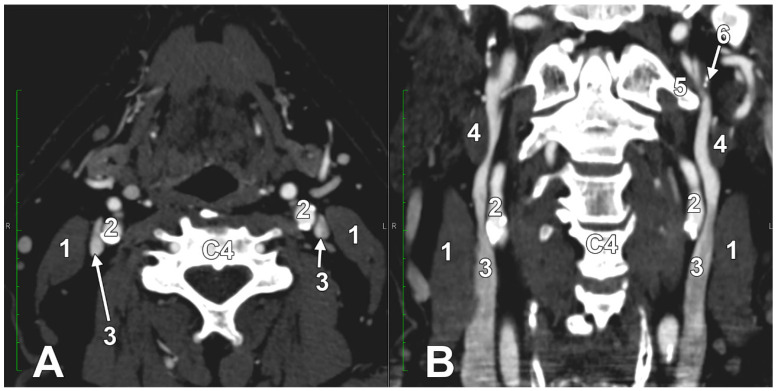
Bilateral triple compressions of the internal jugular veins (IJVs). The upper sites of compression are between the stylohyoid chain and the transverse processes of the atlas, respectively. The middle sites of compression are at C2, deep to the digastric muscle. The lower compression sites are, bilaterally, between the sternocleidomastoid muscle and the atheromatous internal carotid artery. (**A**) Axial slice at the level of the C4 vertebra, viewed inferiorly; (**B**) coronal slice through the IJVs, viewed anteriorly. 1. Sternocleidomastoid muscle; 2. internal carotid artery; 3. IJV; 4. digastric muscle; 5. transverse process of the atlas; 6. ossified keratohyal.

Cases have shown that a tortuous ICA can externally compress the IJV, resulting in IJV stenosis. Symptoms are often nonspecific and may be misattributed to other conditions, resulting in a delayed diagnosis. Although two cases of bilateral IJV stenosis by tortuous ICAs were reported previously [166], this condition seems to be overlooked. While the specific literature on atheromatous carotid artery compression of the IJV, shown in Figure 7, Figure 8 and Figure 9, appears limited, the concept is anatomically and pathophysiologically plausible because of the following: (a) anatomical proximity—the CCA, ICA, and IJV run nearby within the carotid sheath; (b) mass effect—atheromatous plaques can cause arterial wall thickening and luminal irregularity; and (c) vascular remodelling—atherosclerotic changes can alter vessel geometry. A dynamic pseudo-nutcracker jugular compression may be considered when the IJV is pinched between the SCM and the CCA (Figure 9). Other studies did not search for specific muscle details related to IJV compression [40,133,174], although these may add, at different cervical levels, to a nutcracker compression between the atlas and the SP (Figure 9 and Figure 10).

The SCM and omohyoid (OMH) muscles were not listed by Jayaraman et al. (2012) as potential extrinsic compressive structures of the IJV [45], although extracranial venous compression of the IJV by the SCM and OMH muscles can lead to head rotation-induced vertigo, headaches, or even hydrocephalus [33,34,175]. With the mouth open and during deep inspiration, a significant increase in the IJV surface was found above the OMH; therefore, the role of compression of the IJV by the OMH muscle is supported [176]. The resulting modifications in cerebral venous hemodynamics can thus be affected by yawning [176].

SCM/OMH compression is typically induced with ipsilateral head turning and flexion, and is usually bilateral with concurrent compressions that include stylohyoid and thoracic outlet syndrome [33]. Therefore, symptom relief from vertigo and headache can be achieved through the muscular release of IJV compression, which can be accomplished surgically or with the use of a neurotoxin [33,34]. An entrapment of the IJV between the SCM and OMH muscles was found in a Ménière patient and was resolved by physiotherapy treatment for muscular compression, primarily focused on the SCM and OMH [142]. A fibrotic and short OMH intermediate tendon may determine a stricture of the IJV [137].

When an atypical OMH compresses the IJV, partly or entirely, it may be surgically transected [137,143,177]. Transient narrowing of the left IJV due to extrinsic compression from the SCM with leftward head rotation was objectified and solved surgically in a case [178]. The dynamic compression of the IJV by a hypertrophied hyoid bone and thyroid cartilage was also reported [149].

Although different studies involve the SP in IJV compression, an unossified segment of the SHC, tensed between two ossified segments (Figure 6), may also be compressive. The severe stenosis of the IJV at C4–5, with a 4 mm Hg trans-stenosis gradient, was found to be due to the extrinsic compression of the IJV between the SCM muscle and the carotid axis [32]. The IJV may also be entrapped between an atypically attached SCM and the CCA [143].

### A Long Inferior Petrosal Sinus May Also Enter into the Nutcracker

The IPS is a paired dural venous sinus in the posterior cranial fossa that drains the cavernous sinus into the jugular bulb. It receives an inflow from the auditory structures and the brainstem [179,180]. Endovascular access to the IPS has diagnostic and therapeutic utility for diverse conditions involving the cavernous sinus and sellar regions [179]. The IPS can be used for the embolisation of the cavernous dural arteriovenous fistulas or venous plexuses of the skull base [181]. Bilateral IPS sampling is an essential means for the diagnosis and differential diagnosis of pituitary microadenomas [181]. A long extracranial IPS courses along the IJV to empty into it at a lower level [181]. This extracranial extension of the IPS may be regarded as an accessory IJV [182]. It was demonstrated that a long or aberrant IPS may also be used for transvenous embolisation for endovascular management [183].

Different reports of duplicated IJVs were previously published [184,185,186,187,188]. In a complete IJV duplication, there are two separate IJVs instead of the typical single vein [189]. Incomplete, high bilateral duplications were also reported [190]. If the IJV duplication is regarded as a bifurcation of the vein, with each branch having a separate connection to the subclavian vein, and a fenestration of the IJV is regarded as a bifurcation that reunites proximal to the subclavian vein [187], a high duplication of the IJV can be equally regarded as a long IPS (accessory IJV) adjacent to a main IJV, and ending into that one, or vice versa. This ambiguous interpretation is logical, as the upper connections of the two IJVs are not carefully verified. The IJVs should continue the sigmoid sinus, and the IPS should connect to the cavernous sinus. Confusing a true duplicate IJV with a long IPS will lead to an erroneous endovascular route, directing the approach towards the sigmoid sinus rather than the cavernous one.

We present here a case with bilateral long IPSs (Figure 7 and Figure 8). The novelty lies in the fact that both the long IPS and the IJV were pinched within the C1/SP nutcracker. This is because, on the one hand, the stenosis of the IJV determines different neurological deficits, discussed elsewhere in this paper, and, on the other hand, a compressed IPS could not be of use as an endovascular passage when it is compressed into a nutcracker. Specific documentation of IPS compression between the SP and atlas appears to be either very rare in the literature or potentially underreported. During an anatomical study of the archived angioCT file of a 63-year-old male case, bilateral long IPSs were found. The right one was 1.25 cm long, and the left one was 5.15 cm long. The right one had a diameter of 0.2 cm. The opposite one had a diameter of 0.32 cm. The right IPS was joined beneath the jugular foramen by the lateral condylar vein. The left IPS descended antero-medially to the left IJV. Both these veins were applied and compressed on the anterior side of the transverse process of the atlas. The two veins continued anterolaterally to the transverse process of the axis, and at 0.41 cm below that process, the IPS ended into the IJV. On both sides, the ICAs crossed anteriorly to the IPSs to enter the carotid canals. Therefore, the anatomic route of the IPS, the long IPS-to-IJV confluence site, and the diameter of the IPS are relevant in clinical procedures, such as cavernous sinus sampling, which requires catheterisation of both IPSs. Knowledge of the venous anatomy, including variants of the IPS and its branches, is crucial for the diagnosis and treatment of parasellar lesions.

## 8. Clinical Anatomy

The clinical conditions associated with IJV compression are (1) chronic cerebrospinal venous insufficiency, a controversial condition where IJV stenosis or compression is thought to impair cerebral venous drainage; it has been studied in relation to multiple sclerosis, though the connection remains debated [15,20]; (2) intracranial hypertension: IJV compression can contribute to impaired cerebral venous outflow and may lead to increased intracranial pressure; (3) postural effects: head position significantly affects IJV patency; rotation, flexion, or extension of the neck can compress the IJV; and (4) supine vs. upright positioning affects venous drainage patterns. A possible link may exist between impaired IJV outflow and certain central nervous system disorders, such as multiple sclerosis, Alzheimer’s disease, leukoaraiosis, migraine, transient global amnesia, transient monocular blindness, Parkinson’s disease, and Meniere’s disease [5]. The anatomo-clinical correlations should, however, be considered with caution.

Different clinical manifestations may be related to a stenotic IJV, but with different prevalence: tinnitus (60.5%), tinnitus cerebri (67.4%), headache (48.8%), dizziness (32.6%), neck discomfort (39.5%), dry or puffy eyes (37.2%), anxiety or depression (39.5%), sleep disturbance (60.5%), hearing disorder (39.5%), visual disorder (39.5%), and subjective memory decline (30.2%) [5]. Vertigo, memory disturbances, head noises, ear pain, hearing impairment, cognitive fatigue, brain fog, and even cranial haemorrhage were reported in such cases [191].

Cerebral venous outflow from the brain is not fully understood [191]. A simple compression of the IJV may be benign, while stenosis of the IJV can be pathological. Evidence is emerging that the presence of surrounding venous collaterals and white matter hyperintensities may assist in distinguishing whether an IJV compression is benign or pathological [191].

A case of IJV compression by an ESP was reported, presenting with 3 years of positional headache associated with decreased vision when reading and while turning the head to the right or left [126]. The patient also reported pulsatile low-frequency tinnitus, and papilledema was noted on the physical examination [126]. After styloidectomy, the visual fields normalised, and the symptoms disappeared [126]. Jugular venous compression by the ESP will become symptomatic in the context of bilateral styloid compression, dominant venous system compression, or contralateral venous occlusion [136]. The venous congestion resulting from the compression of the IJV by the SP might lead to the development of a de novo cerebral cavernous malformation [192].

Chronic or positional IJV compression (often from anatomical structures like the SP or muscles) can cause headaches, tinnitus, insomnia, and, in severe cases, intracranial hypertension [45,147,148]. Acute IJV compression increases cerebral blood flow velocity and pial artery pulsation, which may have implications for small vessel disease if prolonged [160,193,194,195].

### 8.1. Cannulation of the Internal Jugular Vein

Cannulation of a central vein is a widespread procedure. The IJV is commonly used for this purpose. This procedure is used for various medical reasons. It can be used for haemodynamic monitoring [2]. It enables the delivery of blood products and drugs [2]. Total parenteral nutrition is another indication [2]. The procedure also allows haemodialysis [2]. It helps with the management of perioperative fluids [2]. The most significant target area for central venous line placement is the lower portion of the right IJV [23]. Cannulation of the IJV, being performed at the base of the neck, eliminates the risk of pneumothorax, major arterial bleeding, and failure [2]. While right-handedness typically correlates with an enlarged right-sided IJV, the potential for a larger left-sided IJV should not be overlooked [196]. Therefore, ultrasound-guided central venous cannulation is advised to prevent complications and minimise multiple needle insertion attempts [196].

Complications may occur in more than 15% of patients undergoing central venous catheterisation [197]. Complications can be mechanical, thromboembolic, and infectious [197]. Anatomical variations, particularly small IJV diameters, may play a role in the genesis of mechanical complications [197]. Knowledge about the agenesis of the IJV is essential to avoid failures or complications of the central venous cannulation [54]. The overall failure rate of IJV catheterisation was 10.1% for experienced operators and 19.4% for inexperienced ones [198].

Cannulation of the right IJV is preferable to the left IJV, but when the right IJV is not available for central venous access, the left IJV may be used [2,50,199]. In such cases, it is advisable to document whether the targeted IJV is anatomically compressed. Rotation or extension of the head does not significantly change the size of the IJV [17]. Variations in position and the relationship between the IJV and the carotid arteries may lead to inadvertent artery puncture, which could be disastrous during central venous access [18]. As it descends in the neck, the IJV gradually moves from the lateral side of the CCA to anterior to it, so the percentage overlap of the IJV and CCA gradually increases [18]. Compared with the left side at the same transverse level, the distance between the CCA and the IJV is wider on the right side, and the right IJV is wider [18]. In 11/200 patients, the IJV was found medial to the CCA at one or more transverse scan levels [18]. The angle between the IJV and CCA was significantly smaller in elderly patients [18]. An imaging study found different IJV-to-CCA relations: the IJV was lateral to the CCA in 85.2% of cases, anterior to it in 12.5%, medial to it in 1.1%, and posterior to it in 1.1% [199]. In a different study, the IJV was found anterior and lateral to the CCA in 92% of cases, which corresponds to the typical anatomical descriptions [200].

**Figure 11:**
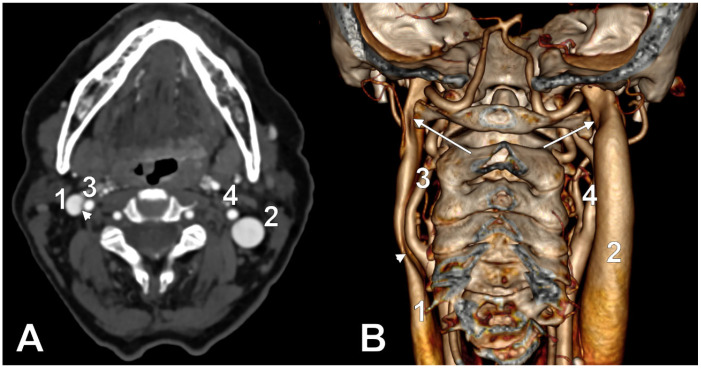
Bilateral asymmetry of the jugular–carotid interrelations. (**A**) Axial slice above the carotid bifurcation, viewed superiorly. (**B**) Three-dimensional volume rendering, posterior view. 1. Non-dominant left internal jugular vein; 2. dominant right internal jugular vein; 3. left internal carotid artery; 4. right internal carotid artery. The internal jugular veins are bilaterally compressed by the transverse processes of the atlas (arrows). The left internal carotid artery compresses the internal jugular vein (arrowhead).

A right dominant IJV is presented in Figure 11. One may observe that the IJV course, as related to the carotid axis, is bilaterally asymmetrical; the left IJV is lateral to the carotid axis, while the right IJV is posterolateral to the carotid axis. Seemingly, the bilateral symmetry of the IJV/carotid topography was consistently overlooked in previous studies [199]. An ultrasonographic study on 80 critically ill patients found that in 62.5% of cases, asymmetric IJVs [44]. The right IJV was dominant in 68% of cases, and the left IJV in 32% [44]. The IJVs were studied using transverse sections, 15 mm above the cricoid cartilage [44].

### 8.2. Stenosis of the Internal Jugular Vein

Chronic cerebrospinal vascular insufficiency is a serious disease process, often accompanied by abnormal intracranial pressure caused by cerebral venous outflow insufficiency. IJV stenosis has been associated with chronic cerebrospinal vascular insufficiency [201].

Previous studies have employed somewhat ambiguous criteria for defining venous stenosis; however, the generally accepted definition involves a reduction of more than 50% in the venous cross-sectional area at one point compared to the vessel’s maximum diameter anywhere along its length [15]. This measurement can be obtained through either conventional venography (using vessel diameter) or ultrasound examination (using cross-sectional area) [15].

Distinctive grades of IJV compression were used with different imaging techniques to evaluate IJV compression: (1) stenotic segment narrowing of ≥50% with respect to the proximal adjacent jugular vein segment; (2) at least one abnormal collateral vessel ≥50% of the maximal diameter of the adjacent IJV or at least two abnormal collateral vessels <50% of the maximal diameter of the adjacent IJV; (3) IJV stenosis secondary to the compression from the C1 lateral mass with or without the SP; and (4) IJV stenosis with unexplained non-focal neurological deficits or other symptoms [40].

Moreover, venous anatomy and wall properties are known to fluctuate considerably in response to internal pressure, volume status, and flow dynamics [15]. The venous system’s substantial capacitance and compliance enable accommodation of major volume or flow shifts with minimal pressure changes [15]. Under conditions of low intraluminal pressure or when external pressure exceeds internal pressure, venous walls collapse and the circular cross-section flattens [15] (Figure 10). These basic hemodynamic principles, along with the naturally high compliance of venous structures, make stenosis evaluation challenging [15]. Thus, luminal calibre variations in these highly compliant vessels might reflect normal physiologic flow patterns rather than true stenotic disease [15].

Imaging features can be used to differentiate between physiologic IJV slenderness and pathologic IJV stenosis [201]. This can be extended to IJV compressions. Key imaging characteristics that define IJV stenosis include several distinctive features. These are localised narrowing accompanied by abnormal collateral veins, cloud-like white matter hyperintensities, and a size discrepancy between the IJV’s transverse diameter and the jugular foramen calibre [201].

A study on patients with cerebral venous outflow disorders found that most commonly, ipsilateral rotation results in ipsilateral IJV stenosis and gradient development at C4–6 and contralateral stenosis and gradient appearance in the contralateral IJV at C1, with stenosis and gradient development in bilateral IJVs at C1–3 bilaterally during chin flexion [202]. Of 89 patients, 93.3% developed at least moderate dynamic stenosis of at least one IJV, 69.7% developed either severe or occlusive stenosis during both rightward and leftward rotation, and 81.8% developed severe or occlusive stenosis with head flexion [202]. It is therefore reasonable to conclude that the ipsilateral rotation of the head or chin flexion in cases with compressed IJVs may severely increase IJV stenosis.

A recent study found no significant correlation between IJV stenosis in the J3 segment of the IJV and intracranial pressure [36]. The J3 segment of the IJV displays right dominance [36]. Stenosis in the J3 segment yields a diminished outflow in the IJV and an augmented outflow in the vertebral veins [36]. When the bilateral flow volume in the J3 segment exceeds 425 mL/min, it may denote intracranial hypertension [36].

#### Multiple Stenotic Segments of the Internal Jugular Vein

**Figure 12:**
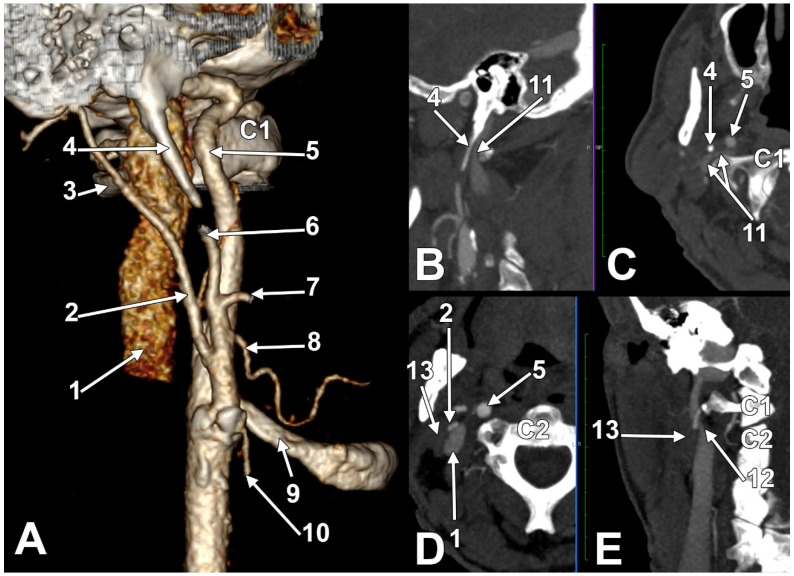
Compressions of the internal jugular vein (IJV) at C1 and C2. Right side. (**A**) Three-dimensional volume rendering, anterolateral view. (**B**) Sagittal slice through the styloid process and C1 transverse process, viewed medially. (**C**) Axial slice through the atlas. (**D**) Axial slice through the axis. (**E**) Coronal slice through the second site of compression of the IJV, viewed anteriorly. 1. IJV; 2. occipital artery; 3. transverse process of the atlas; 4. styloid process; 5. internal carotid artery; 6. external carotid artery; 7. facial artery; 8. lingual artery; 9. greater hyoid horn; 10. superior thyroid artery; 11. compressed IJV, on the anterior side of the transverse process of the atlas; 12. compressed IJV, deep to the digastric muscle; 13. digastric muscle.

Multiple levels of compression or stenosis affecting the IJV may be found at different anatomical points along its course (Figure 12). Therefore, when a compression site of the IJV is found, other compressions should not be excluded but carefully documented. Although the J3 segment of the IJV is usually involved, stenoses may also be found in the J2 and/or J1 segments of the vein [5], and, therefore, the IJV must be investigated entirely for compressions. While the concept of sequential IJV compression is recognised clinically, specific studies focusing on “sequential distortions” as a distinct entity are limited. Most research focuses on single-level compression mechanisms. The compression levels of the IJV can be listed as follows: (1) superior level: SP, C1 transverse process, SCM insertion area; (2) middle level: SCM, fascial planes, cervical spine elements; and (3) inferior level: OMH, thoracic inlet, clavicular area. The clinical implications of sequential distortions of the IJV may include the following: (a) Hemodynamic effects: Each compression point creates additional resistance; thus, the effect on cerebral venous drainage may be cumulative; they may cause more severe symptoms than single-level compressions. (b) Diagnostic challenges: Multiple compression points may mask each other on imaging; thus, dynamic evaluation is needed to assess all levels. Doppler studies must examine the entire IJV course. (c) Treatment considerations: Multilevel intervention may be required. Surgical planning must address all compression points; there is a risk of incomplete symptom resolution if levels are missed.

### 8.3. Thrombosis of the Internal Jugular Vein

IJV thrombosis is an infrequent condition that comprises 1.5% of deep venous thromboses overall and 45.3% of upper limb deep vein thromboses [128]. The leading risk factors include central line placement, cancer, and ovarian hyperstimulation syndrome [128]. Pokeerbux et al. (2020) reported the initial case of IJV thrombosis potentially attributed to venous entrapment between the SP and C1 transverse process [128].

IJV thrombosis may lead to pulmonary embolism [203]. In approximately 20% of cases of pulmonary embolism, the source of emboli cannot be identified, and it may be speculated that IJV thrombosis in a stylo-jugular syndrome may be responsible for some of these cases [203].

Guan et al. (2021) reported a case of cerebral venous sinus thrombosis in the lateral sinus in a patient with bilateral compression of the IJVs due to an overgrown left lateral mass of the atlas, as well as arteriosclerosis and expansion of the right ICA [135].

### 8.4. Solving the Decompression of the Internal Jugular Vein

Scerrati et al. (2021) analysed 13 papers reporting 149 patients with extrinsic, styloidogenic IJV compression at the level of C1 [133]. All patients were medically treated, in most cases with anticoagulants [133]. Endovascular treatment was performed in 33.6% of these cases, and stenting was performed in 30.2% of them [133]. Surgery was performed in 36.9% of the cases and consisted of styloidectomy (27.5%), C1 tubercolectomy (1.3%), and styloidectomy and C1 tubercolectomy (8.1%) [133]. Combined styloidectomy and stenting was reported in 18.8% of these cases [133]. The decompressive step must be taken before stenting is performed [157]. Bolognese et al. (2024) reported a case with symptomatic bilateral compression of the IJVs, primarily attributable to the transverse processes of the atlas [148]. Decompression was achieved by bilateral resection of the C1 tubercles [148]. The C1 transverse process resection could be added to a styloidectomy if the SP also compresses the IJV [204]. Different surgical approaches may be used for styloidectomy: transcervical, postauricular, or extreme lateral infracondylar [204]. 

Stenting may be considered for focal IJV stenosis with significant pressure gradients, primarily when caused by vascular compression [166]. In nutcracker syndromes of the IJV, primary stenting may not only be ineffective but may actually exacerbate the outflow obstruction [111]. To achieve optimal jugular flow restoration, a combination of endovascular treatment and surgery is necessary [133]. This is because the endovascular procedure alone may not be effective if the mechanical compression by bony structures is not relieved [133]. Surgery alone may not be sufficient to completely re-expand the chronically compressed vein [133]. Care must be taken when skeletonising the IJV near the ESP [205].

Surgical decompression via the extreme lateral infracondylar approach has shown promising results, with significant improvement in IJV stenosis and clinical symptoms in most patients. In a recent case series, 8 out of 14 patients experienced significant symptom relief, and imaging confirmed improved venous flow in the majority of patients [156]. Styloidectomy has a reported success rate of 79%, higher than that of angioplasty or stenting (66%), with minimal complications in surgical cases [206]. However, some patients may require additional interventions, such as stenting, if symptoms persist [32,156].

While generally safe, surgical IJV decompression can result in complications, such as cranial nerve paresis and wound infection, though these are relatively rare [156]. Stenting, when used, carries a higher complication rate and risk of symptom recurrence, emphasising the need for careful patient selection and surgical expertise [32]. Most neurointerventionists should not perform IJV stenting unless they have experience with these patients and understand the technical nuances, including IJV anatomy, which can maximise benefit and minimise risk [32].

In a case with extrinsic compression of the IJV by a hypertrophic hyoid bone and thyroid cartilage, venous decompression was achieved by the resections of the posterior part of the greater hyoid horn and the superior horn of the thyroid cartilage [149].

## 9. Conclusions

The jugular nutcracker can also involve and compress an elongated inferior petrosal sinus (IPS), which serves as an accessory IJV. This represents a discovery of critical importance for precise preoperative anatomical assessment.

The IJV may display multiple pathways, including fenestrations and duplications across different segments. Its size and bilateral symmetry vary unpredictably. Styloid process geometry differs between patients, meaning the SP may not always compress the IJV. Other nearby anatomical structures may be responsible for IJV compression instead.

Therefore, thorough IJV documentation is essential before any surgical or interventional procedures. Clinicians have to distinguish carefully between proven findings and hypothetical associations.

## Data Availability

The datasets used and analysed during the current study are available from the corresponding author upon reasonable request.

## Abbreviations

The following abbreviations are used in this manuscript:

CCA	common carotid artery
CT	computed tomography
EA	Eagle’s syndrome
ECA	external carotid artery
ESP	elongated styloid process
HJB	high jugular bulb
IAC	internal acoustic canal
ICA	internal carotid artery
IJV	internal jugular vein
IPS	inferior petrosal sinus
JB	jugular bulb
JBD	jugular bulb diverticulum
JNS	jugular nutcracker syndrome
OA	occipital artery
OMH	omohyoid muscle
PAA	posterior auricular artery
PSC	posterior semicircular canal
SCM	sternocleidomastoid muscle
SHC	stylohyoid complex
SP	styloid process
